# Cardiac biomarkers in term newborns with common pathological conditions during the first 24 hours postpartum

**DOI:** 10.11613/BM.2025.030702

**Published:** 2025-08-15

**Authors:** Helena Karlović, Marjana Jerković Raguž, Ivanka Mikulić, Vinka Mikulić, Vajdana Tomić

**Affiliations:** 1 Clinic for Children Diseases, University Clinical Hospital Mostar, Mostar, Bosnia and Herzegovina; 2 Department of Neonatology, Clinic for Children Diseases, University Clinical Hospital Mostar, Mostar, Bosnia and Herzegovina; 3 School of Medicine, University of Mostar, Mostar, Bosnia and Herzegovina; 4 Department of Laboratory Diagnostics, University Clinical Hospital Mostar, Mostar, Bosnia and Herzegovina; 5 Faculty of Pharmacy, University of Mostar, Mostar, Bosnia and Herzegovina; 6 Department of Obstetrics and Gynecology, University Clinical Hospital Mostar, Mostar, Bosnia and Herzegovina

**Keywords:** cardiac biomarkers, neonatal jaundice, perinatal infection, term neonate, transient neurological abnormalities

## Abstract

**Introduction:**

Cardiac biomarkers may help diagnose and monitor different neonatal conditions, but their concentrations are still underexplored in common pathologies diagnosed within the first day. This study compared N-terminal pro b-type natriuretic peptide (NT-proBNP), high sensitivity troponin I (hs-TnI), creatine kinase (CK), and its isoenzyme creatine kinase-myocardial band (CK-MB) concentrations and activities, measured within the first 24 hours (h) postpartum, between the healthy term neonates and those with jaundice, perinatal infection, transient neurological abnormalities (TNA), and heart ultrasound abnormalities.

**Materials and methods:**

The study included 100 term newborns, whose cardiac biomarkers’ concentrations were determined from the serum within 24 h postpartum on the Alinity ci analyzer (Abbott, Chicago, USA). The Mann-Whitney and Kruskal-Wallis tests, performed in SPSS Statistics v. 25.0 (IBM Corp., Armonk, USA), were used to test the significance of differences between the study groups, with P < 0.05 indicating significance.

**Results:**

Within first 24 h postpartum healthy newborns had significantly higher CK activities compared to those with jaundice (P = 0.047), perinatal infection (P = 0.012), or combination of both (P = 0.017). Lower CK activities were demonstrated in perinatal infection compared to TNA (P = 0.041). Other biomarkers’ concentrations did not differ between the study groups. No significant differences were found in cardiac biomarkers’ concentrations regarding gender or heart ultrasound findings.

**Conclusions:**

During the first 24 h postpartum, only CK activities differed between healthy newborns and those with the common pathologic conditions, being lower in the newborns with jaundice and/or infection. Analogous differences were present between newborns with infection and those with TNA.

## Graphical abstract

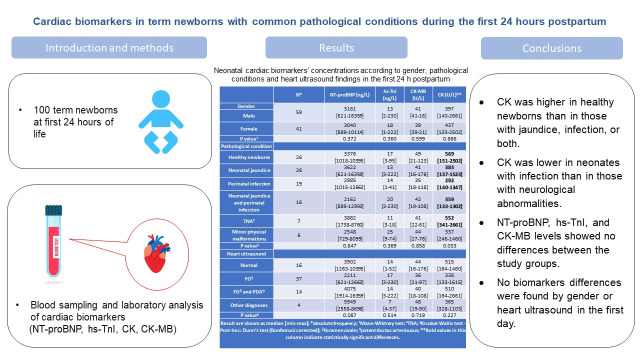

## Highlights

• Creatine-kinase (CK) activity was higher in healthy newborns than in those with jaundice, infection, or both

• CK was lower in neonates with infection than in those with neurological abnormalities

• N-terminal pro b-type natriuretic peptide (NT-proBNP), high sensitivity troponin I (hs-TnI) concentrations, and CK- myocardial band activity showed no differences between the study groups

• No biomarkers differences were found by gender or heart ultrasound in the first day

## Introduction

In neonates, the transition from fetal to the neonatal hemodynamics involves unique physiological changes: closure of natural heart shunts, a decrease in pulmonary artery pressure, and an increase in systemic circulatory pressure (*1*). The transition in perinatal circulation and the shift of blood flow from the placenta to the lungs elevate ventricular volume and pressure load. As B-type natriuretic peptide (BNP) and its inactive by-product, N-terminal pro b-type natriuretic peptide (NT-proBNP) are indicators of cardiac volume expansion and pressure overload, they are consequently synthesized and released from myocardial cells into the circulation after birth (*2*, *3*). Therefore, within the first 24 hours (h) of life, NT-proBNP concentration may be elevated, with a tendency for a rapid decline during the first week, with gradual decrease over time and stabilization during childhood (*4*).

High sensitivity troponin I (hs-TnI), creatine kinase (CK), and its isoenzyme creatine kinase-myocardial band (CK-MB) are well known markers of myocardial damage. The concentrations of these cardiac biomarkers are commonly elevated at birth, which can be attributed to possible transient hypoxia associated with the process of delivery, combined with the transition of the cardiorespiratory system to postnatal life. The decline in hs-TnI, CK, and CK-MB concentrations and activities over time follows a pattern similar to NT-proBNP, with their concentrations decreasing in the first days of life until they reach stable lower concentrations that persist throughout adult life (*4*, *5*).

In most healthy newborns, the transition from fetal to neonatal circulation, as described above, along with the closure of the *ductus arteriosus* and *foramen ovale* (FO), is expected within the first 48 h of life (*6*). However, in some newborns, these openings remain persistently open, resulting in patent *foramen ovale* (PFO) and patent *ductus arteriosus* (PDA) (*7*). Neonates with PDA have tendency to have significantly higher NT-proBNP and troponin concentrations which is associated with an increase in oxygen demand of the cardiac cells and positively correlates to hemodynamic severity of PDA (*4*, *8*). It was observed that low NT-proBNP concentration or a decrease in its concentration, could indicate closure of the *ductus arteriousus*, while higher concentrations were associated with later PDA (*4*).

As previous research shows, various pathological conditions of the neonatal age are proven to influence the values ​​of neonatal cardiac biomarkers. It has been shown that perinatal asphyxia (PA) and its complications, such as hypoxic-ischemic encephalopathy (HIE), bronchopulmonary dysplasia, and persistent pulmonary hypertension of the newborn, lead to a significant increase in NT-proBNP concentrations. Cardiovascular dysfunction is a common outcome in term infants following PA, as it causes injury of many organs, including the heart (*9*). As it is known, hs-TnI, CK and CK-MB are indicators of cardiac injury, and higher concentrations of these biomarkers have been also established in neonates with HIE (*10*, *11*). Studies have shown a tendency for NT-proBNP concentrations to increase in neonates with pulmonary hypertension or cardiomyopathies, neonatal sepsis, and those with various forms of congenital heart disease (CHD) (*4*, *12*-*15*).

The role of hs-TnI in the diagnosis and monitoring of CHD remains conflicting based on current research findings. However, it has been observed that hs-TnI concentrations are significantly higher in severe cases of respiratory distress compared to healthy groups. No significant differences between healthy neonates and those with CHD or among different CHD groups have been established when it comes to CK-MB activities. However, CK-MB activities were notably higher in CHD neonates who underwent surgical correction during the neonatal hospitalization period compared to those who didn’t (*4*). Furthermore, there is evidence suggesting that CK-MB could serve as a predictor of mortality in neonatal shock (*16*). While existing literature describes variations in cardiac biomarker values concerning pathological conditions in neonates, gender-based differences have generally not been significant in prior studies (*3*, *17*).

As it’s seen above, current knowledge indicates that cardiac biomarkers could be utilized to diagnose and monitor certain cardiac and non-cardiac pathological conditions (*16*, *18*, *19*). However, the available literature does not sufficiently address the relationship between cardiac biomarkers’ concentrations and certain pathological conditions commonly encountered within the 24 h postpartum, such as neonatal jaundice, perinatal infection, transient neurological abnormalities (TNA), and heart ultrasound abnormalities (*2*, *10*, *20*, *21*). Considering the aforementioned, and described variations in cardiac biomarkers’ concentrations in the early neonatal age, determining their concentrations during this period seemed necessary in order to make a distinction between expected cardiac biomarkers’ concentrations in the mentioned pathological conditions, and the concentrations that could be potentially associated with more serious pathological conditions in neonate within the first 24 h postpartum (*4*, *5*, *9*, *10*, *12*-*16*). This study compared NT-proBNP, hs-TnI, CK, and CK-MB concentrations and activities, measured within the first 24 h postpartum, between the healthy term neonates and those with jaundice, perinatal infection, TNA, and heart ultrasound abnormalities. We consider that term neonates diagnosed with described neonatal pathologies tend to have higher cardiac biomarkers’ concentrations compared to healthy term neonates in the first 24 h of life.

## Materials and methods

### Subjects

This study was conducted in University Clinical Hospital Mostar (UCHM) from January 2023 to March 2024. The study encompassed term neonates in first 24 h of life who were born at a gestational age (GA) from 37+0/7 to 41+6/7 weeks at the Clinic for Gynecology and Obstetrics of UCHM. Preterm newborns, newborns with chromosomal abnormalities, and resuscitated newborns with poor Apgar scores (< 8) were excluded from this study. A preterm newborn is considered to be a newborn GA less than 37+0/7 weeks. It involved only neonates whose parents consented to participate in the research, confirmed by signing a written informed consent. In addition to healthy neonates, the study included those with various pathological conditions, with diagnoses made based on clearly defined guidelines. The diagnosis of neonatal jaundice was defined according to the National Institute for Health and Care Excellence (NICE) guidelines from 2016, which implied a total serum bilirubin concentration > 100 µmol/l in neonates immediately after birth, > 125 µmol/L at 6 h, > 150 µmol/L at 12 h, > 175 µmol/L at 18 h, and > 200 µmol/L at 24 h of life (*22*). Transient neurological abnormalities considered transient deviations in newborns’ muscle tone, such as hypertonia, hypotonia, and variable tone (*23*). Minor physical malformations included preauricular appendages, syndactyly, and low-set ears (*24*). Exclusion criteria for pregnant women were any pathological conditions that have been proven to affect fetal growth and development such as hypertension, diabetes mellitus, autoimmune disease, *etc.* (*25*). Mothers’ and newborns’ data were taken from the hospital information system and delivery register.

All procedures performed in the study involving human participants were in accordance with the ethical standards of the institutional research committee and with the 1964 Helsinki Declaration and its later amendments or comparable ethical standards. The study was approved by the Ethics Commission University Clinical Hospital Mostar (call number 1071/22, dated 03.07.2022).

### Methods

In this study venous blood samples were used for laboratory analysis, as they are previously taken for routine laboratory tests (required by a neonatologist during the first 24 h of a newborn’s life), as part of the post-delivery examination. After the routine analyses requested by neonatologists were completed, the remaining serum was used for the analysis of cardiac biomarkers in this study. No additional blood samples were taken for analyses during the study. Median cubital vein or dorsal hand veins were most frequently used for venipuncture. Venous blood samples were collected in Micro sample tubes with gel (Sarstedt, Nümbrecht, Germany; volume 1.3 mL) with Micro needles 23 G (Sarstedt, Nümbrecht, Germany). The collected samples were centrifuged at 2320xg for 10 minutes on Eppendorf Centrifuge 5804R (Germany) and stored at - 80 °C until analysis. Concentrations of NT-proBNP and hs-TnI, as well as CK and CK-MB activities were measured on Alinity ci analyzer (Abbott, Chicago, USA). The chemiluminescent microparticle immunoassay (CMIA) was used to determine NT-proBNP and hs-TnI concentrations. Activity of CK was determined using a kinetic ultraviolet (UV) test, and CK-MB using an enzyme immunoinhibition test.

Echocardiography was performed during the first 24 h of the newborn’s life, when the neonatologist observed a heart murmur or any other clinical sign that could indicate the presence of cardiac pathology (*26*, *27*). A specified examination has been performed on the General Electric Vivid T8 (GE HealthCare, Chicago, USA).

### Statistical analysis

The Mann-Whitney and Kruskal-Wallis tests, performed in SPSS Statistics v. 25.0 (IBM Corp., Armonk, USA), were used to test the significance of differences between the study groups, with P < 0.05 indicating significance. *Post-hoc* testing included pairwise comparisons using Dunn’s method with Bonferroni correction. Results are expressed as absolute frequency, median, minimum and maximum.

## Results

This study included total of 100 term newborns, encompassing 26 healthy newborns, and 74 newborns with pathological conditions, primarily jaundice or perinatal infection (Table 1). Heart ultrasound was performed on 71 newborns, and abnormalities were established in 55 of them, most commonly FO (Table 1). Regarding heart ultrasound, the category “other diagnoses” included newborns with diagnoses of ventricular septal defect (VSD) and atrial septal defect (ASD).

**Table 1:** Distribution of participants based on pathological conditions and heart ultrasound findings.

	N*
**Pathological conditions**	
Neonatal jaundice	26
Perinatal infection	19
Neonatal jaundice and perinatal infection	16
TNA	7
Minor physical malformations	6
**Heart ultrasound**	
Normal	16
FO	37
FO and PDA	14
Other diagnoses	4
*absolute frequency. TNA - transient neurological abnormalities. FO - *foramen ovale*. PDA - patent *ductus arteriousus*.	

Regarding laboratory findings, a significant difference was observed for CK activities (Table 2). Healthy newborns had significantly higher CK activities compared to those with jaundice (P = 0.047), perinatal infection (P = 0.012), and neonates who had both jaundice and perinatal infection (P = 0.017) within the first 24 h postpartum. Additionally, it was found that neonates with perinatal infection have significantly lower CK activities compared to neonates with TNA (P = 0.041) in the first day of life. Other biomarkers’ concentrations did not differ between the study groups. Differences based on gender, as well as heart ultrasound findings within the first 24 h of neonatal life, were not significant for any biomarker (Table 2).

**Table 2:** Neonatal cardiac biomarkers’ concentrations and activities ​​according to gender, pathological conditions and heart ultrasound findings in the first 24 h postpartum

	N*	**NT-proBNP** **(ng/L)**	**hs-TnI** **(ng/L)**	**CK-MB** **(U/L)**	**CK** **(U/L)**	
**Gender**						
Male	59	3181 (621-16,359)	13 (2-230)	41 (41-16)	397 (140-2661)	
Female	41	3040 (889-10,114)	18 (1-222)	39 (39-21)	437 (133-2502)	
P value		0.372	0.360	0.599	0.866	
**Pathological condition**						
Healthy newborns	26	3376 (1018-10,395)	17 (3-95)	49 (21-123)	569 (151-2502)	
Neonatal jaundice	26	3622 (621-16,359)	13 (3-222)	40 (16-176)	383 (137-1523)	
Perinatal infection	19	2985 (1015-12,663)	14 (1-41)	35 (18-118)	292 (140-1347)	
Neonatal jaundice and perinatal infection	16	2162 (889-12,993)	20 (3-230)	42 (19-108)	359 (133-1302)	
TNA	7	3882 (1738-8760)	11 (3-18)	41 (22-61)	552 (341-2661)	
Minor physical malformations	6	2548 (729-8099)	25 (9-74)	44 (27-76)	337 (246-1460)	
P value		0.847	0.369	0.858	0.033	
**Heart ultrasound**						
Normal	16	3902 (1263-10,395)	14 (1-52)	44 (16-176)	515 (164-1460)	
FO	37	2211 (621-12,663)	17 (3-230)	36 (21-97)	338 (133-1615)	
FO and PDA	14	4075 (1914-16,359)	14 (3-222)	40 (18-108)	510 (164-2661)	
Other diagnoses	4	5949 (2559-8698)	7 (4-37)	48 (19-90)	365 (328-1103)	
P value		0.087	0.514	0.719	0.227	
*absolute frequency. Result are shown as median (min-max), P value is assessed by Mann-Whitney (gender) or Kruskal-Wallis test. TNA - transient neurological abnormalities. NT-proBNP - N-terminal pro b-type natriuretic peptide. hs-TnI - high sensitivity troponin I. CK - creatine kinase. CK-MB - creatine kinase-myocardial band. FO - *foramen ovale*. PDA - patent *ductus arteriousus*.						

## Discussion

Overall, this study emphasizes that healthy neonates have higher CK activities compared to neonates with jaundice, perinatal infection, or both jaundice and perinatal infection within the first 24 h postpartum. At the same time, neonates with perinatal infection have lower CK activities compared to those with TNA in the first day of life. Concentrations of NT-proBNP, hs-TnI, and CK-MB activity did not differ between the neonates with different pathologic conditions or in comparison with the healthy neonates. Additionally, there was no difference in the concentrations of any cardiac biomarker based on gender or heart ultrasound findings within the first 24 h of neonatal life.

Liu *et al*. reported significantly increased serum CK activities in neonatal jaundice patients within the bacterial infection group, with a positive correlation to inflammatory markers (*20*). Their study included only newborns with jaundice and compared those with and without infection, ultimately suggesting that bacterial infection might contribute to myocardial dysfunction in this population. In contrast, our results did not show a significant difference in CK activities between jaundiced newborns with or without perinatal infection. These differing outcomes may be attributed to several factors, including the larger sample size and the broader age range (up to 28 days) in the study by Liu *et al*., compared to our study, which was limited to the first 24 h postpartum.

Elevated concentrations of unconjugated bilirubin may contribute to myocardial injury in neonates through multiple mechanisms, including alterations in membrane permeability, direct interference with mitochondrial function, oxidative stress, and tissue hypoxia (*28*). Despite these potential effects of hyperbilirubinemia on cardiac muscle, no increase in NT-proBNP, hs-TnI, CK, or CK-MB concentrations was observed in this study group. Regarding hs-TnI, our findings are consistent with those of Taksande *et al*., who observed no significant difference in troponin I concentrations or cardiac structure between healthy neonates and those with unconjugated hyperbilirubinemia (*21*). Although the age range in their study was broader, the comparable results may indicate that neonatal jaundice is not necessarily associated with myocardial injury in the early postnatal period.

Conducted study also demonstrates higher CK activities in healthy newborns compared to those with perinatal infection. Unfortunately, the relationship between this cardiac biomarker and perinatal infection has not yet been investigated, which opens up opportunities for further research.

The recent studies have shown that NT-proBNP production is enhanced by inflammatory and endothelial mediators as part of cardiovascular dysfunction occurrence in neonatal sepsis (*15*). As such, they suggest that NT-proBNP could find its purpose as an indicator of neonatal sepsis and its outcome (*2*, *13*-*15*). However, in neonates with perinatal infection, higher NT-proBNP concentrations were not observed in this study. During the early stages, inflammation associated with sepsis causes a slight rise in NT-proBNP concentrations, likely as a result of the release of small amounts of BNP stored in granules. However, if the condition progresses to septic shock and myocardial dysfunction, this increase becomes more pronounced due to the *de novo* synthesis of the peptide and rapid gene expression (*14*). Since NT-proBNP concentrations in this study were measured within the first 24 h of life, this could potentially explain the absence of a marked increase in NT-proBNP in examined neonates.

Although this study found that neonates with perinatal infection have lower CK activities compared to neonates with TNA within the first 24 h of life, comparable results have not been documented in previous studies. However, it has been shown that neonates with neurological deviations may have elevated CK activities, especially in first days of life (*10*). Numerous studies have also obtained that neonates with neurological deficits also tend to have elevated NT-proBNP, hs-TnI, and CK-MB concentrations (*9*, *11*, *29*). Conversely, this study did not detect elevated concentrations of the mentioned biomarkers during the first day of life in newborns with neurological deficits.

Contrary to previous findings, conducted study did not reveal differences in cardiac biomarkers’ concentrations between neonates with normal heart ultrasound and those with ultrasound abnormalities (FO, PDA, VSD, ASD). According to many other studies, including meta-analysis by Teixeira *et al*., higher NT-proBNP concentrations were recorded in neonates with PDA compared to the group in which PDA was naturally closed, including positive correlation to hemodynamic severity of PDA (*2*, *4*, *18*). Vaisbourd *et al.* also demonstrated higher troponin concentrations in preterm neonates who have hemodynamically significant PDA compared with nonsignificant PDA (*8*).​Nevertheless, the lack of differences in cardiac biomarkers’ concentrations presented in the conducted study are not uncommon. Even studies that used the same research kits obtained somewhat different results. Although, such an occurrence has not been fully explained, it is believed that the age of newborns after birth plays a crucial role in the diagnosis of neonatal CHD and that it can be attributed to significant changes in NT-proBNP concentrations within the first few days of life (*2*). Additional studies are needed to better clarify the role of neonatal NT-proBNP concentrations in assessing the presence and severity of CHD.

Furthermore, the study indicates that there were no differences in cardiac biomarkers’ concentrations between genders, whether or not pathological conditions or heart ultrasound abnormalities were present. Similar results are shown in the existing literature, with the emphasis that these studies often used broader timeframes within the neonatal period for measuring biomarker values or included fewer participants compared to this study (*3*, *17*).

A key limitation of the study is the overall small sample size, particularly when compared to previous studies involving larger number of neonates (*2*-*4*, *6*, *21*, *30*). This limitation is further emphasized by the very small number of participants in certain subgroups, which should be taken into account when interpreting the results.

In conclusion, this study suggest that, within the first 24 h of life, CK activities may vary depending on neonatal health status, being lower in newborns with jaundice, perinatal infection, or both, compared to healthy controls, as well in newborns with infection compared to those with TNA, while other cardiac biomarkers remained consistent across the study groups. These results emphasize the importance of timing in cardiac biomarkers’ evaluation and highlight the need for further studies to clarify the early postnatal dynamics of cardiac biomarkers in various neonatal conditions.

## Data Availability

The data generated and analyzed in the presented study are available from the corresponding author on request.
